# Genetic characterization of porcine reproductive and respiratory syndrome virus from Eastern China during 2017–2022

**DOI:** 10.3389/fmicb.2022.971817

**Published:** 2022-10-13

**Authors:** Lujia Zhou, Yang Yang, Qiqi Xia, Zhixin Guan, Junjie Zhang, Beibei Li, Yafeng Qiu, Ke Liu, Donghua Shao, Zhiyong Ma, Xiaodu Wang, Jianchao Wei

**Affiliations:** ^1^College of Animal Science and Technology and College of Veterinary Medicine of Zhejiang A&F University, Hangzhou, Zhejiang, China; ^2^Chinese Academy of Agricultural Sciences, Shanghai Veterinary Research Institute, Shanghai, China

**Keywords:** Eastern China, genetic characterization, ORF5, PRRSV, GP5 glycoprotein, Nsp2

## Abstract

Porcine reproductive and respiratory syndrome (PRRS) is an immunosuppressive disease caused by PRSS virus (PRRSV). PRRSV mainly causes reproductive disorders in pregnant sows and respiratory diseases in piglets. Recently, it has emerged as one of the most important diseases of the pig industry across the globe. In this study, we have collected 231 samples from differently sized pig farms in Eastern China from 2017 to 2022 to investigate the epidemic characteristics of the disease. All samples were screened by RT-PCR and analyzed further using *Nsp2* and *ORF5* genes. The result showed that the positive rate of PRRSV was 24% (54/231). Phylogenetic analysis (13 positive samples) revealed that all isolates belonged to genotype 2, and they were mainly distributed in four lineages (i.e., lineage 1, 3, 5, and 8). Nsp2 is the most variable protein among all PRRSV NSPs, several isolates from this study had amino acid deletions within Nsp2 compared to that of strain VR-2332. The major structural protein glycoprotein (GP5) protein is encoded by ORF5. Epitope analysis of the 13 isolated strains and additional reference strains revealed that all 13 strains had some mutations on the decoy epitope, the primary neutralizing epitope, T cell epitopes, and B cell epitopes. This study showed that the prevalent PRRSV strain in Eastern China was still HP-PRRSV, while the proportion of NADC30-like and NADC34-like strains have increased. This study further enriches the epidemiological data of PRRS in Eastern China and provides a theoretical basis for vaccine development and prevention and control of the disease across the region.

## Introduction

Porcine reproductive and respiratory syndrome (PRRS) is an acute infectious disease caused by the PRSS virus (PRRSV). PRRS first appeared in Europe and North America in the 1980s (Wang et al., 2019). Subsequently, the disease quickly spread worldwide and caused huge economic losses in the swine industry. The clinical symptoms of PRRS are mainly caused by reproductive disorders in sows and respiratory diseases in piglets (Fang et al., 2022).

Porcine reproductive and respiratory syndrome virus (PRRSV) is a member of the Arteriviridae family in the order Nidovirales (Adams et al., 2017). PRRSV is an enveloped, positive single-stranded RNA virus (Chen et al., 2020). The PRRSV genome is approximately 15 kilobases (kb) in length and contains at least 10 open reading frames (ORFs) and two untranslated regions at the 5′ and 3′ ends of the genome (Zhang et al., 2022). ORF1a and ORF1b encode at least 16 nonstructural proteins (Nsps) which are related to replication (Fang and Snijder, 2010; Fang et al., 2012). ORF2a, ORF2b, ORF3–5, ORF5a, ORF6, and ORF7 encode eight viral structural proteins (Wu et al., 2001). Among the NSPs, Nsp2 is the largest PRRSV nonstructural protein which contains a highly variable region. Because this region usually observed mutations, insertions, or deletions, Nsp2 could be a unique pointer for monitoring the genetics and evolution of PRRSVs (Saenglub et al., 2020). ORF5 encodes the major structural protein glycoprotein 5 (GP5). Because of the high variability of ORF5, GP5 is usually used for phylogenetic analyses and classification of PRRSV isolates (Murtaugh et al., 2010). GP5 is an important immunogenic protein, which contains several primary neutralizing epitopes (PNEs) and N-linked glycosylation sites (NGSs; Ostrowski et al., 2002). PNEs can produce non-neutralizing antibodies against GP5 and tend to delay the production of neutralizing antibodies against PRRSV (Stoian and Rowland, 2019). The deletion of NGSs in GP5 alters the susceptibility of the virus to the host’s neutralizing antibodies through a phenomenon known as “glycan shielding,” thereby evading vaccine-induced immune responses (Rupasinghe et al., 2022).

Based on the distinct genetic and antigenic diversity, PRRSV isolates were divided into two major genotypes: the European genotype (PRRSV-I) and the North American genotype (PRRSV-II; Kuhn et al., 2016). The prototype representative strains were Lelystad and VR-2332, respectively (Fang et al., 2022; Zhang et al., 2022). These two genotypes share only 60% nucleotide identity (Shi et al., 2010a). PRRSV-I has been divided into four genetically distinct subtypes (Fitzgerald et al., 2020). Based on a comprehensive of ORF5 sequences, PRRSV-II has been classified into nine lineages. Most of the PRRSV-II were identified in the United States and distributed in North America and Asia (Paploski et al., 2019, 2021; Kikuti et al., 2021). Although PRRSV-I and PRRSV-II co-exist in the Chinese swine herds, PRRSV-II is predominant in China (Gao et al., 2017; Guo et al., 2018). The prevalent PRRSV-II strains in China were clustered into four lineages: lineage 1, lineage 3, lineage 5 (sublineage 5.1), and lineage 8 (sublineage 8.7). In 1996, the first isolated PRRSV strain CH-1a was reported in China, which belongs to lineage 8 (Valicek et al., 1997). CH-1a was recognized as an ancestor strain of classical PRRSV in China, and its presence suggested that PRRSV was widespread in China. Then in 2006, an undefined swine endemic with high incidence and mortality broke out in Jiangxi Province, China. It was later determined that the pathogen of this endemic was a PRRSV variant with a characteristic unique discontinuous deletion of 30 amino acids in Nsp2, which was named highly pathogenic PRRSV (HP-PRRSV) belonging to sublineage 8.7 (An et al., 2007; Tong et al., 2007). Since then, HP-PRRSV gradually became the main PRRSV type causing epidemics in mainland China (Peng et al., 2017; Liang et al., 2019). HP-PRRSV presently also circulating in most Southeast Asian countries including Laos, Vietnam, Cambodia, Bhutan, and India (Rajkhowa et al., 2022). The representative sublineage 5.1 strain BJ-4 was isolated in 1997, showing 99.6% genome homology with VR2332 (Peng et al., 2017; Liang et al., 2019). In 2010, the widely occurring lineage 3 strains QYYZ and GM2 were reported in Southern China (Shi et al., 2010b). Lineage1 is known to have a long epidemic history since it initially appeared in Canada in the 1990s. Since the initial outbreak in North America in the early 2000s, its representative strain is MN184 (Ramos et al., 2022). In 2013, a new cluster of lineage1.8 named NADC30 spread from Canada to the United States (Yu et al., 2021). At the same time, NADC30-like PRRSV isolates were reported in Henan Province, China. The detection rates of NADC30-like strains in China have gradually increased. PRRSV strain NADC30, which has a discontinuous 131-amino-acid deletion in Nsp2 and is moderately virulent, was first isolated in the United States (Brockmeier et al., 2012). In 2014, the sublineage 1.5 representative strain NADC34 started to emerge in the United States and caused large abortion outbreaks and high mortality rates in piglets (van Geelen et al., 2018). Isolate NADC34 has a continuous deletion of 100 amino acids in Nsp2. From 2015 to 2017, various NADC34-like (PRRSV 1-7-4) strains were identified in Peru, and one whole genome of NADC34-like strain was reported from South Korea (Ramirez et al., 2019; Kim et al., 2022). At the same time, two novel PRRSV NADC34-like isolates (LNWK96 and LNWK130) were reported in Liaoning Province, China (Zhang et al., 2018). Since then, different regions of China (Heilongjiang, Henan, Fujian, and Jiangsu) have reported NADC34-like PRRSV strains (Zhang et al., 2018; Liu et al., 2019; Xie et al., 2020). Epidemiological investigations suggest that NADC34-like PRRSV has become endemic in China. PRRSV has high gene mutation and recombination frequencies, and in the epidemic process, new strains have developed. These factors have led to increased diversification and prevalence of clinical strains, impeding disease prevention (Wenhui et al., 2012; Lu et al., 2015).

In this study, we aimed to reveal the prevalence and genetic evolution of PRRSV during 2017–2022 in different regions of Eastern China. We have also identified various PRRSV strains and spotted critical amino acid variation among them based on ORF5 and Nsp2 proteins. Our study provides a theoretical basis for further monitoring of PRRSV variants in China.

## Materials and methods

### Sample collection

A total of 231 samples (lung or serum) were collected from different scale pig farms between 2017 and 2022 across Eastern China, including Jiangsu, Zhejiang, Anhui, and Shandong provinces (Supplementary Table 1). Some pigs showed significant clinical symptoms of PRRSV infection, including fever, labored breathing, abortions, and mummified fetus. Lungs sample were ground by a grinder and diluted in sterilized phosphate-buffered saline. Homogenized samples were centrifuged for 5 min at 5,000 rpm and stored at 4°C until RNA extraction. Serum samples were used directly for subsequent experiments.

### RNA extraction, PCR amplification, and sequencing

Genomic RNA was extracted from 200 μl of viral stocks using the TIANamp Virus RNA/DNA Kit (TIAN GEN, Beijing, China) according to the manufacturer’s instructions. The viral cDNA was synthesized by reverse transcription with the Evo M-MLV RT Premix (ACCURATE BIOLOGY, Hunan, China) as recommended by the manufacturer and stored at −80°C until further processing. The ORF5 region and Nsp2 hypervariable regions were amplified by RT-PCR and subjected to DNA sequencing. The primers for ORF5 and Nsp2 sequences were listed in Table 1 (Xie et al., 2020). The amplification reactions were carried out in 50 μl PCR reaction volume containing 25 μl of 2 × Hieff PCR Master Mix (Yeasen Biotechnology, Shanghai, China), 4 μl primers, 2 μl template, and 19 μl distilled water. After pre-denaturation for 5 min at 95°C, 35 cycles of amplification were performed (95°C for 30 s, 58°C for 15 s, and 72°C for 45–60 s), followed by a final extension step at 72°C for 7 min.

**Table 1:** Primers used for Porcine reproductive and respiratory syndrome virus (PRRSV) detection in this study.

Primer	Sequence (5′–3′)	Product size (bp)
ORF5-F	GGCGACCGTTTTAGCCTGTCTT	735
ORF5-R	ATCATTATTGGCGTGTAGGTG	
Nsp2-F	TTGATTGGGATGTTGTGCTTC	681/774/984/1074
Nsp2-R	CAATGATGGCTTGAGCTGAGT	

The PCR products were analyzed by electrophoresis using 1% agarose gel under UV light. The positive bands were sliced and the gel was purified using the Gel Extraction Kit (Omega, United States) and cloned into the pMDTM18-T vector (TAKARA, Japan). The plasmids were sequenced by Qingke Company (Shanghai, China).

Thirteen positive samples (randomly selected) were inoculated into primary porcine alveolar macrophages (PAMs; obtained from specific pathogen-free piglets) or MARC-145 cells, respectively. The inoculated cells were cultured in an incubator at 37°C and 5% CO_2_ until cytopathic effects (CPE) were observed. The virus was harvested by repeated freezing and thawing for further analysis, and stored at −80°C.

### Phylogenetic analysis, amino acid alignment, and glycosylated analysis

The multiple sequence alignments of nucleotide and amino acid were performed by clustalW method using DNASTAR version 7.0 software (DNASTAR Inc., Madison, WI, United States; Lole et al., 1999). Detailed information on isolated PRRSV strains and reference strains are shown in Tables 2, 3. The phylogenetic trees were constructed by MEGA 7.0 (AZ, United States) with the neighbor-joining method from 1,000 bootstrap replicates for alignment, using multiple sequences of PRRSV available in GenBank (Chen et al., 2017). The asparagine (N)-linked glycosylation sites of GP5 protein were predicted using the NetNGlyc 1.0 Server [NetNGlyc-1.0—redirect (dtu.dk); Gupta and Brunak, 2002].

**Table 2:** Information of 13 PRRSV isolates from Eastern China in this study.

Strain name	Province (State)	Accession no.	Year
AH-BZ-2021-04	Anhui (Bozhou)	ON357667	2021
JS-KS-2021-12	Jiangsu (Kunshan)	ON357668	2021
ZJ-JX-2017-04	Zhejiang (Jiaxing)	ON357678	2017
JS-LYG-2017-12	Jiangsu (Lianyungang)	ON357669	2017
AH-BB-2018-01	Anhui (Bengbu)	ON357666	2018
JS-LYG-2018-06	Jiangsu (Lianyungang)	ON357671	2018
JS-LYG-2018-05	Jiangsu (Lianyungang)	ON357670	2018
JS-LYG-2018-07	Jiangsu (Lianyungang)	ON357672	2018
JS-NJ-2018-08	Jiangsu (Nanjing)	ON357673	2018
JS-NJ-2021-12	Jiangsu (Nanjing)	ON357674	2021
JS-NJ-2022-01	Jiangsu (Nanjing)	ON357675	2022
ZJ-HZ-2022-02	Zhejiang (Hangzhou)	ON357677	2022
SD-QL-2021-11	Shandong (Linyi)	ON357676	2021

**Table 3:** Information about 29 PRRSV reference strains used in the sequence and phylogenetic analysis.

Strain name	Country (Province)	Year	Accession no.
Lelystad virus	Netherlands	1993	M96262
EuroPRRSV	USA	1999	AY366525
IA/2014/NADC34	USA	2014	MF326985
IA/2015/NADC35	USA	2015	MF326986
LNWK130	China (Liaoning)	2017	MG913987
FJ0908	China (Fujian)	2018	MK202794
PRRSV-ZDXYL-CHina-2018-1	China (Heilongjiang)	2018	MK453049
JS2021NADC34	China (Jiangsu)	2021	MZ820388
HLJZD30-1902	China (Heilongjiang)	2019	MN648055
NADC30	USA	2008	JN654459
WHU5	China (Hubei)	2015	KU523366
MN184A	USA	2002	DQ176019
CHsx1401	China (Shanxi)	2014	KP861625
HENAN-XINX	China (Henan)	2014	KF611905
JL580	China (Heilongjiang)	2014	KR706343
QYYZ	China (Guangdong)	2011	JQ308798
GM2	China (Guangdong)	2011	JN662424
FJFS	China (Fujian)	2012	KP998476
VR-2332	USA	1995	U87392
BJ-4	China (Beijing)	1996	AF331831
RespPRRSV MLV	United States	1994	AF066183
CH-1a	China (Beijing)	1996	AY032626
CH-1R	China (Beijing)	2008	EU807840
HB-1(sh)/2002	China (Hebei)	2002	AY150312
TJ	China (Tianjin)	2006	EU860248
HUN4	China (Hunan)	2006	EF635006
JXA1	China (Jiangxi)	2006	EF112445
JXA1-P80	China (Beijing)	2008	FJ548853
JXA1-P170	China (Beijing)	2012	JQ804986

## Results

### RT-PCR analysis of clinical samples

In this study, 231 samples were collected from various pig farms across four provinces of Eastern China and were screened for PRRSV through RT-PCR. 24% (54/231) of the tested samples were found to be positive for the PRRSV. A total of 13 different ORF5 gene sequences and Nsp2 gene hypervariable region sequences were obtained after excluding identical sequences. The isolated sequences were deposited to GenBank (details summarized in Table 2) and further analysis was performed.

### Sequence determination, phylogenetic analyses and amino acid alignment of Nsp2

**Figure 1:**
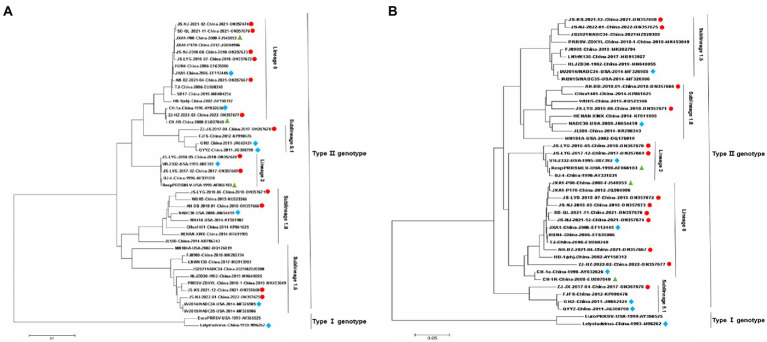
Phylogenetic trees of 13 Porcine reproductive and respiratory syndrome virus (PRRSV) isolates and 29 reference PRRSV isolates based on the *Nsp2* and *ORF5* gene sequences of these isolates. **(A)** Nsp2 nucleotide; **(B)** ORF5 nucleotide. The represent sequences of different PRRSV strains are indicated with blue diamonds. The new PRRSV isolates identified in this study are labeled with red circles and PRRSV vaccine strains are indicated with green triangles.

Nsp2 is the most variable protein among all PRRSV NSPs, and is responsible for viral genetic evolution and pathogenicity (Yu et al., 2020; Zhu et al., 2022). Here, we have constructed a phylogenetic tree based on Nsp2 sequences. The results revealed that all 13 isolated strains belonged to the North American genotype and are further divided into four lineages (Figure 1A). Sequence alignments indicated that nucleotide identities of the 13 isolates ranged between 77.7–99.9, 76.1–99.3, and 76.1–99.2% when compared with the representative strains, i.e., VR-2332, CH-1a, and JXA1, respectively (Table 4). However, when compared with strains QYYZ, NADC30, and NADC34, the nucleotide similarity was seen between 73.9–91.8, 74.5–95.3, and 74.1–97.3% (details are summarized in Table 4).

**Table 4:** Nsp2 and ORF5 genomic nucleotide identity between the 13 isolates and PRRSV reference strains.

Strain name	NADC34		NADC30		QYYZ		VR2332		JXA1		CH-1a	
	Nsp2	ORF5	Nsp2	ORF5	Nsp2	ORF5	Nsp2	ORF5	Nsp2	ORF5	Nsp2	ORF5
JS-KS-2021-12	97.3	96.5	78.5	87.6	74.5	84.7	77.8	86.7	77.7	85.9	78.8	86.7
JS-NJ-2022-01	97.2	96.0	78.7	87.4	74.2	84.6	77.7	86.6	77.3	85.6	78.1	86.4
AH-BB-2018-01	77.9	86.9	95.3	92.2	74.8	84.2	78.9	84.6	76.7	84.1	76.9	85.4
JS-LYG-2018-06	77.5	87.2	94.5	93.0	73.9	82.9	78.6	84.7	76.1	85.2	76.1	86.4
ZJ-JX-2017-04	74.1	84.2	74.5	84.9	91.8	92.4	79.3	84.9	83.5	86.2	83.5	86.7
JS-LYG-2017-12	78.4	87.7	80.5	85.4	79.9	83.4	99.9	98.8	84.8	88.9	87.3	91.5
JS-LYG-2018-05	78.3	87.1	80.6	85.7	80.0	83.6	99.9	98.3	85.0	89.1	87.5	91.5
JS-LYG-2018-07	7.8	86.2	77.1	84.9	83.2	83.1	84.6	87.9	98.9	97.8	92.5	93.9
JS-NJ-2018-08	77.7	86.2	77.0	85.2	83.1	82.9	84.7	88.4	99.2	98.0	92.6	94.0
JS-NJ-2021-12	77.7	86.2	77.1	85.2	83.1	82.9	84.6	88.7	99.0	98.2	92.3	94.2
ZJ-HZ-2022-02	78.9	86.7	78.4	85.6	85.3	82.8	87.2	85.6	93.0	90.5	99.3	90.5
SD-QL-2021-11	77.7	86.2	77.1	85.2	83.1	82.8	84.7	88.7	99.1	99.2	92.4	94.5
AH-BZ-2021-04	78.0	86.2	77.2	85.7	83.4	83.3	85.0	88.4	99.2	97.8	92.8	93.9

**Figure 2:**
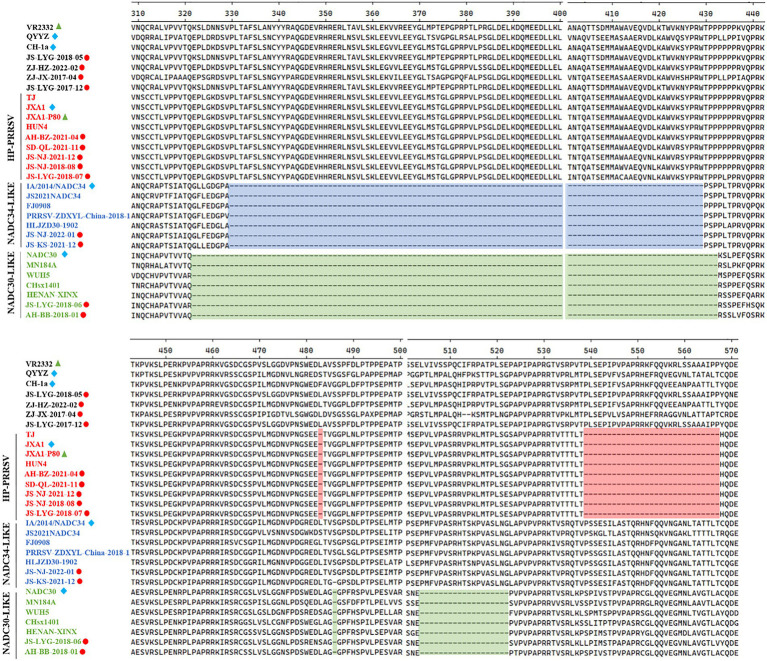
Alignment of the Nsp2 amino acid sequences of the 30 PRRSV strains. The 30-amino-acid discontinuous deletion of HP-PRRSVs is shown in red shadow. The 100-amino-acid continuous deletion of NADC34-like PRRSVs is shown in blue shadow. The 131-amino-acid discontinuous deletion of NADC30-like PRRSVs is shown in green shadow.

Several isolates from this study had amino acid deletions within Nsp2 when compared to strain VR-2332. However, JS-NJ-2022-01 and JS-KS-2021-12 isolates had a continuous 100 amino acids deletion, which were similar to the NADC34-like strain (Figure 2). JS-LYG-2018-06 and AH-BB-2018-01 isolates had a discontinuous deletion of 131(111 + 1 + 19) amino acids, which were characteristic of NADC30-like strains (Figure 2). Apart from this, the five isolates belonging to HP-PRRSV strain in this study have a discontinuous deletion of 30 (1 + 29) amino acids (Figure 2).

### Sequence determination and phylogenetic analyses of ORF5

A phylogenetic tree was constructed to compare the ORF5 sequences of the 13 isolated PRRSV strains with 29 reference strains gotten from the GenBank database (Table 3). The results revealed that 13 isolates from this study are further divided into five lineages, i.e., lineage 1 (1.5,1.8), lineage 3, lineage 5 (5.1) and lineage 8, which is similar to the results obtained from Nsp2 (Figure 1B). Among the 13 isolated strains, 6 strains were closely related to the lineage 8, sharing 90.5 to 99.2% nucleotide identity. Four strains, i.e., JS-KS-2021-12, JS-NJ-2022-01, AH-BB-2018-01, and JS-LYG-2018-06 belongs to sublineage 1.5 and sublineage 1.8. JS-KS-2021-12 and JS-NJ-2022-01 shared 96.5 and 96.0% nucleotide identity with the lineage 1.5 representative strain NADC34. AH-BB-2018-01 and JS-LYG-2018-06 shared 92.2 and 93.0% nucleotide identity with the sublineage 1.8 representative strain NADC30. JS-LYG-2017-12 and JS-LYG-2018-05 were clustered into sublineage 5.1, with the representative strain VR2332 sharing 98.8 and 98.3% nucleotide identity. Respectively. Only one strain, i.e., ZJ-JX-2017-04, shared high nucleotide identity (92.4%) with the lineage 3 representative strain QYYZ (details are summarized in Table 4). Strain QYYZ was identified as potential recombinant strain between a field strain and a vaccine strain of RespPRRS MLV.

### Amino acid mutation analysis of GP5

Multiple amino acid sequence alignment revealed that all GP5 proteins of the 13 isolated strains and the reference strains consisted of 200 amino acids, encoded by 603 nucleotides. No deletions and additions were found, but substitutions were frequently observed. The 13 strains all shared > 88% amino acid sequence identity with reference strains.

**Figure 3:**
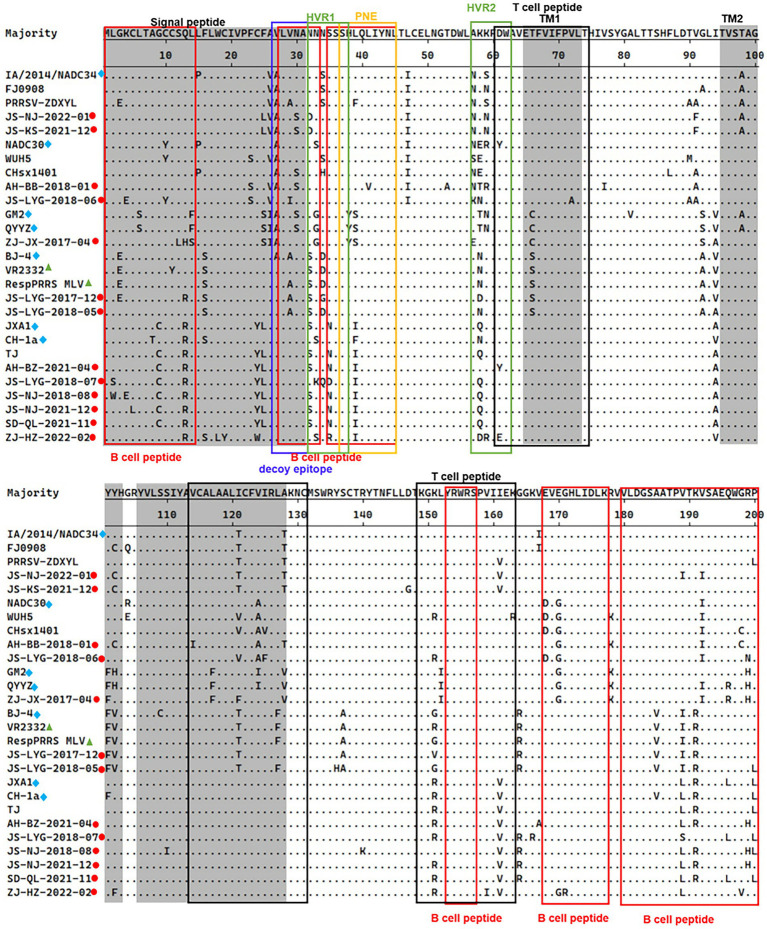
Alignment of full-length GP5 amino acid sequences of 13 positive PRRSV strains and 14 reference PRRSV strains. The represent PRRSV strains are indicated with blue diamonds, the new PRRSV isolates identified are labeled with red circles and PRRSV vaccine strains are indicated with green triangles. The grey areas represent signal peptide and transmembrane regions (TM). Red boxes indicated B-cell epitopes and black boxes indicated T-cell epitopes. Primary neutralizing epitope (PNE) was shown in blue box and the decoy epitope was shown in yellow box. Hypervariable regions (HVR) were shown in green box.

The 13th and 151st positions in the GP5 protein are related to viral virulence. R^13^ and R^151^ are characteristic of virulent strains. At the position 13th, 7 out of 13 strains had an R^13^ residue. Only strain ZJ-JX-2017-04 had an H^13^ residue and all other isolated strains had a Q^13^ residue. At the 151st position, 6 out of 13 strains had an R^151^ residue, strain JS-LYG-2018-05 had a G^151^ residue, strain JS-LYG-2017-12 had a V^151^ residue and all other isolated strains had a K^151^ residue (Figure 3).

### Functional domain analysis of the GP5 protein

The PRRSV-II GP5 protein harbors 1 signal peptide, 2 hypervariable regions, 3 transmembrane regions, and 11 epitopes (Figure 3; Zhou et al., 2018). The epitopes include a decoy epitope, PNE, T cell epitopes, and B cell epitopes.

Amino acid analysis of GP5 protein epitopes revealed that the variation of the GP5 protein was mainly concentrated in T cell and B cell epitopes (Figure 3). Furthermore, different lineages had particular amino acid mutations. Compared to sublineage 5.1, lineage 3, and sublineage 8.7, sublineage 1.5 and sublineage 1.8 had less substitutions in epitopes. Sublineage 1.5 harbors two unique amino acid mutations in T cell epitopes, including T^121^ and T^128^ (Figure 3). Sublineage 1.8 harbors five unique amino acid mutations in T cell and B cell epitopes, including N/S^33^, E/D^168^, G^170^, I/V^121^, and A^124^ (Figure 3). Lineage 3, sublineage 5.1, and sublineage 8.7 had some substitutions at T cell and B cell epitope positions (Figure 3).

Analysis of the PNE and the decoy epitope of GP5 revealed that four strains had the same PNE sequence as the RespPRRS MLV vaccine isolate (PNE sequence: ^37^SHLQLIYNL^45^). Relative to the RespPRRS MLV vaccine isolate, six strains had an L^39^ → I^39^ substitution in the PNE (Figure 3). ZJ-JX-2017-04 had two substitutions (H^38^ → Y^38^ and L^39^ → S^39^) in the PNE, which were the same as in the lineage 3 representative strains QYYZ and GM2 (Figure 3). Two other strains had unique amino acid substitutions in the PNE sequence. Strain JS-LYG-2018-06 had a deletion at the 37th position, and strain AH-BB-2018-01 had an ^41^L → ^41^V substitution (Figure 3). The 13 strains collectively displayed some substitutions within the decoy epitope (^27^VLVNA^31^) of GP5. Residues 27 and 29 were particularly polymorphic. Four strains had an A^27^ residue and two strains had an A^29^ residue (Figure 3). Additionally, strain JS-LYG-2018-06 had a particular I^29^ residue (Figure 3). Three strains had a low-frequency amino acid substitution (S^30^) within the decoy epitope (Figure 3).

### N-linked glycosylation site analysis of the GP5 protein

GP5 has multiple N-glycosylation sites, which is likely related to immune evasion by shielding neutralizing antibodies (Akter et al., 2021; Zhu et al., 2022). There are five N-linked glycosylation sites in the amino acid position N^30^, N^32/33/34^, N^35^, N^44^, and N^51^ of the GP5 glycoprotein of Betaarterivirus suid2 (Akter et al., 2021). The potential N-glycosylation sites (NGSs) at N44 and N51 were conserved in all isolates, but the potential NGSs located upstream of N44 were relatively variable (Fang et al., 2022). The 13 isolated strains were predicted to have two to five NGSs. In all strains except strain AH-BB-2018-01, N^44^C^45^T^46^ and N^51^G^52^T^53^ had no mutations or deletions and were highly conserved (Table 5). In all strains except strain JS-LYG-2018-07, N^32/33/34^ had no deletion (Table 5). There were 10 strains except JS-NJ-2018-08, JS-NJ-2021-12 and SD-QL-2021-11 had a deletion NGS at N^35^. Eight of 13 strains had one deletion at N^30^ (Table 5).

**Table 5:** Comparation of potential N-glycosylation sites (NGSs) on GP5 protein.

Isolates	Lineage	N-glycosylation sites							NGSs number
		N30	N32	N33	N34	N35	N44	N51	
JS-KS-2021-12	1.5			✔	✔		✔	✔	4
JS-NJ-2022-01	1.5			✔	✔		✔	✔	4
FJ0908	1.5		✔	✔			✔	✔	4
LNWK130	1.5		✔		✔		✔	✔	4
JS2021NADC34	1.5		✔	✔			✔	✔	4
AH-BB-2018-01	1.8			✔	✔		✔		3
JS-LYG-2018-06	1.8			✔	✔		✔	✔	4
WHU5	1.8		✔	✔			✔	✔	4
HENAN-XINX	1.8				✔		✔	✔	3
JL580	1.8				✔		✔	✔	3
ZJ-JX-2017-04	3				✔		✔	✔	3
QYYZ	3				✔		✔	✔	3
GM2	3			✔			✔	✔	3
FJFS	3				✔		✔	✔	3
JS-LYG-2017-12	5.1	✔		✔			✔	✔	4
JS-LYG-2018-05	5.1	✔		✔			✔	✔	4
VR-2332	5.1				✔		✔	✔	2
BJ-4	5.1	✔		✔			✔	✔	4
RespPRRSV MLV	5.1	✔		✔			✔	✔	4
JS-LYG-2018-07	8.7						✔	✔	2
JS-NJ-2018-08	8.7	✔			✔	✔	✔	✔	5
JS-NJ-2021-12	8.7	✔			✔	✔	✔	✔	5
ZJ-HZ-2022-02	8.7				✔		✔	✔	3
SD-QL-2021-11	8.7	✔			✔	✔	✔	✔	5
AH-BZ-2021-04	8.7	✔			✔	✔	✔	✔	5
CH-1a	8.7				✔		✔	✔	3
JXA1	8.7	✔			✔	✔	✔	✔	5
HUN4	8.7	✔			✔	✔	✔	✔	5

## Discussion

PRRS is one of the most devastating swine diseases caused by PRRSV and is broadly distributed across the globe. In this study, we have collected 231 samples from different pig farms across four provinces in Eastern China between the year 2017 and 2022 and screened them for PRRSV. Thirteen PRRSV isolates were obtained from samples and their sequences were deposited to GeneBank for further analysis and characterization. The evolutionary history of PRRSV outbreaks in China is roughly divided into three stages (era). The first stage occurred from 1995 to 2006, when typical strains of PRRSV were dominant (Zhou and Yang, 2010). The second stage existed from the year 2006 to 2012, and strain HP-PRRSV became most prevalent (Li et al., 2011). The third stage started in 2012 and is currently going on, in this stage HP-PRRSV is spotted as the main circulating strain, however, co-circulation of new mutants strains, i.e., NADC30-like, GM2, and NADC34-like have been reported in pig farms from some areas across China (Peng et al., 2017; Liang et al., 2019). According to a survey (Chen et al., 2020) HP-PRRSV strain was found to be the most prevalent (51.57%) PRRSV strain circulating in China between the years 2017 to 2018, and NADC30-like strains also accounted for a large part in PPRSV Soutbreaks (43.30%). Meanwhile, the first NADC34-like PRRSV strain was reported in Liaoning Province, and then some NADC34-like PRRSV strains were reported in different regions of China (Zhang et al., 2018; Liu et al., 2019; Xie et al., 2020). As the ORF5 and Nsp2 coding regions are highly variable, so their genomes are used for PRRSV mutational analysis, virus species determination, and molecular epidemiological characteristics (Cha et al., 2004; Shi et al., 2010a). In this study, two neighbor-joining phylogenetic trees were also constructed and their percentage identities were calculated with other isolated and reference strains based on ORF5 and Nsp2 genomes. All 13 strains were found to have lower nucleotide and amino acid identity with the European strains and higher identity with the North American and Chinese PRRSV strains. Most of the isolated strains were HP-PRRSV, which contain discontinuous deletions of 1 and 29 amino acids in the Nsp2 region. The lineage 1 strains also accounted for a large part. Among all 13 strains, three different strains appeared in the Lianyungang pig farm in Jiangsu previously during the years 2017 and 2018, containing PRRSV classic strains, HP-PRRSV, and NADC30-like strains. The epidemic characteristics from the isolated strains were found consistent with the previous and current PRRSV epidemic situation. We also isolated two NADC34-like strains (i.e., JS-KS-2021-12 and JS-NJ-2022-01) from the Jiangsu province, which contain continuous deletions of 100 amino acids. In late 2021 and early 2022, two NADC34-like strains were isolated from the Jiangsu province by Yuan et al. (2022). Yuan et al. (2022) and Zhu et al. (2022) emphasizing that NADC34-like strains have gradually spread and that they have become a potential endemic strain in China. Therefore, further research into NADC34-like strains is necessary.

GP5 has relevant sites for determining viral virulence and plays a key role in the induction of the immune response (Zhang et al., 2017). The amino acids at positions 13 and 151 can be used to distinguish between virulent and attenuated strains. In this study, five isolated strains containing both amino acids at positions R^13^ and R^151^ were found to be highly virulent. These isolates caused abortion in sows with depression, anorexia, hyperthermia, and dyspnea in piglets. Three isolates having R13 or R151 amino acids were moderately virulent and induced moderate clinical signs (i.e. hyperthermia, anorexia, and moderate respiratory signs). The rest of the strains caused milder clinical symptoms, with elevated body temperature in piglets accompanied by loss of appetite and depression. GP5 has several epitopes, including a decoy epitope, a PNE, T cell epitopes, and B cell epitopes. In this study, we found that most strains had amino acid mutations in T cell and B cell epitopes. The PNE (37–45 aa) and the decoy epitope (27–31 aa) of GP5 play important roles in inhibiting the immune response. Some critical amino acid substitutions in these positions may lead to the ability of the virus to evade recognition by neutralizing antibodies, resulting in the failure of live attenuated cellular vaccine strains (Ostrowski et al., 2002). In this study, eight strains belonging to sublineage 5.1 and lineage8 had the same amino acid in antigenic epitopes compared to their representative strains. Strain ZJ-JX-2017-04, which belongs to lineage 3, had an amino acid mutation (^27^ALVSA^31^ → ^27^ALVNA^31^). Compared to lineage 1 representative strains NADC30 and NADC34, four strains belonging to lineage 1 had some amino acid mutations and deletions. Three strains had a mutation from ^27^ALVNA^31^ to ^27^ALVSA^31^, and strain JS-LYG-2018-06 had a special mutation to ^27^VLINA^31^. There were few amino acid variations in the PNE compared to reference strains. Only strain AH-BB-2018 had a mutation at position 41 from L^41^ to V^41^. These mutations may result in a lack of immune cross-protection between different PRRSV strains.

N-glycosylation of viral proteins is a complex post-translational modification that affects protein folding, viral entry, receptor interactions, immune escape and pathogenesis (Das et al., 2011; Rupasinghe et al., 2022). GP5 contains five NGSs, including N^30^, N^32/33/34^, N^35^,N^44^, and N^51^, which determine the antigenic properties and viral susceptibility to neutralizing antibodies (Akter et al., 2021). The potential NGSs located upstream of N^44^ were relatively variable while N^44^ and N^51^ are highly conserved in United States and European strains. Mutations in the N^44^ residue resulted in non-infectious offspring (Ansari et al., 2006). Relevant studies have confirmed that the expression of N-glycosylated GP5 mutants in PRRSV significantly increased the level of neutralizing antibodies in infected piglets. For the missing glycosylation site, it exposes neutralizing epitopes that are masked by glycosylation, thereby enhancing immunogenicity and susceptibility of GP5 protein to neutralizing antibodies (Vu et al., 2011; Peng et al., 2017). The lack of an N-glycosylation site at N^51^ has been shown to increase the sensitivity of the virus to antibody neutralization and enhance the ability of the virus to establish a cognate NAb response (Vu et al., 2011). The 13 strains isolated in this study were subjected to NGS prediction, and two to five NGSs were predicted. Among them, the AH-BB-2018-01 strain is deleted at N51, and the immune failure caused by this strain may be caused by the reduction of NGS.

Currently, commercial RespPRRS MLV vaccines are widely used for the prevention and control of PRRS in China. However, previous studies and this study indicated that commercial vaccines could not provide complete protection against HP-PRRSV, NADC30-like, and NADC34-like strains. Thus, additional PRRS prevention strategies must be developed.

## Conclusion

In conclusion, we have investigated the epidemiological status of PRRSV in Eastern China between the years 2017 and 2022. We have found that highly pathogenic (HP-PRRSV) remained the predominant circulating PPRSV type in the field. The detection rates of NADC30-like and NADC34-like strains have increased in recent years, accounting for a large proportion of PRRSV. Furthermore, several critical mutations have been spotted within Nsp2 and GP5 proteins of isolated strains which may involve in immune escape. This study facilitates further analysis of PRRSV prevalence.

## Data availability statement

The data presented in the study are deposited in the “National Center for Biotechnilogy Information” repository, accession number “ON357667-ON35767”.

## Author contributions

JW, XW, and ZM conceived the study. LZ, YY, and QX analyzed the data. LZ wrote the manuscript for submission. ZG, JZ, BL, YQ, and KL participated in the design of the study, performed the data collection and analysis, and commented on the manuscript. All authors contributed to the article and approved the submitted version.

## Funding

The study was supported by the Shanghai Agriculture Applied Technology Development Program, China (nos. X2022-02-08-00-12-F01195 and X2021-02-08-00-12-F00770 awarded to JW).

## Conflict of interest

The authors declare that the research was conducted in the absence of any commercial or financial relationships that could be construed as a potential conflict of interest.

## Publisher’s note

All claims expressed in this article are solely those of the authors and do not necessarily represent those of their affiliated organizations, or those of the publisher, the editors and the reviewers. Any product that may be evaluated in this article, or claim that may be made by its manufacturer, is not guaranteed or endorsed by the publisher.
