# Supplementary material for: Genetic characterization of porcine reproductive and respiratory syndrome virus from Eastern China during 2017–2022
Source: Front Microbiol. 2022 Oct 13;13:971817. doi: 10.3389/fmicb.2022.971817 (PMC9606797; doi:10.3389/fmicb.2022.971817)
Supplement: Supplementary file 1 [file Data_Sheet_1.docx]

Supplement Table 1 Positive rate of PRRSV from samples collected in Eastern China from 2017 to 2022

| Strain name | Farm Vaccination Status | Herd Size | Sow abortion rates | Piglet clinical symptoms | Isolation Tissues | RPPSV-positive rates  (positive/total) |
| --- | --- | --- | --- | --- | --- | --- |
| AH-BZ-2021-04 | Yes  (HP-PRRSV Vaccine) | ＞8000 | ～25% | severe breathing difficulties，  cough，high fever | Serum | ~27%  (4/15) |
| JS-KS-2021-12 | Yes  (C-PRRSV Vaccine) | ＞2000 | ～10% | loss of appetite,  depression, fever | Serum | ~28%  (2/7) |
| ZJ-JX-2017-04 | Yes  (C-PRRSV Vaccine) | ＞2000 | ～15% | loss of appetite,  depression, fever | Lung | ~12%  (1/8) |
| JS-LYG-2017-12 | No | ＞10000 | ～30% | medium breathing difficulties，  cough，fever | Lung | ~24%  (6/25) |
| AH-BB-2018-01 | Yes  (HP-PRRSV Vaccine) | ＞5000 | ～20% | loss of appetite,  depression, fever | Serum | ~25%  (4/16) |
| JS-LYG-2018-06 | Yes  (C-PRRSV Vaccine) | ＞10000 | ～25% | medium breathing difficulties，  cough，fever | Serum/  Lung | ~23%  (7/30) |
| JS-LYG-2018-05 | Yes  (C-PRRSV Vaccine) | ＞10000 | ～25% | loss of appetite,  depression, fever | Serum/  Lung | ~25%  (7/28) |
| JS-LYG-2018-07 | Yes  (C-PRRSV Vaccine) | ＞10000 | ～15% | severe breathing difficulties，  cough，high fever | Serum/  Lung | ~30%  （9/30） |
| JS-NJ-2018-08 | Yes  (C-PRRSV Vaccine) | ＞7000 | ～20% | medium breathing difficulties，  cough，fever | Lung | ~20%  （3/15） |
| JS-NJ-2021-12 | Yes  (HP-PRRSV Vaccine) | ＞7000 | ～10% | severe breathing difficulties，  cough，high fever | Serum | ~17%  （3/17） |
| JS-NJ-2022-01 | Yes  (HP-PRRSV Vaccine) | ＞7000 | ～10% | loss of appetite,  depression, fever | Serum | ~27%  （5/18） |
| ZJ-HZ-2022-02 | Yes  (C-PRRSV Vaccine) | ＞2000 | ～10% | severe breathing difficulties，  cough，high fever | Serum | ~16%  （1/6） |
| SD-QL-2021-11 | Yes  (HP-PRRSV Vaccine) | ＞6000 | ～10% | severe breathing difficulties，  cough，high fever | Serum | ~12%  （2/16） |

Supplement Table 2 Critical amino acid substitutions in GP5 function epitopes of various PRRSV genotypes.

| Functional epitopes | Position | Genotypes | | | | |
| --- | --- | --- | --- | --- | --- | --- |
|  |  | Sublineage1.5 | Sublineage1.8 | Lineage3 | Sublineage5.1 | Lineage8 |
| decoy epitope | 27 | A | A | A | V/A | V |
|  | 29 | V | V | V | A | V |
|  | 30 | N | N | S | N | N |
|  | 32 | N | N | N | S | S/N |
| Primary neutralizing epitope | 38 | H | H | Y | H | H |
|  | 39 | L | L | S | L | F/I |
| B cell epitopes | 3 | G | G | G | E/G | G |
|  | 6 | L | L | S/L | L | L |
|  | 8 | A | A | A | A | T/A |
|  | 9 | G | G | G | G | C/G |
|  | 13 | Q | Q | Q | R/Q | R |
|  | 14 | L | L | F/S | L | L |
|  | 29 | V | V | V | A | V |
|  | 30 | N | N | S | N | N |
|  | 32 | N | N | N | S | S/N |
|  | 33 | S | N/S | G | N | N/S |
|  | 35 | S | S | S | S | N/S |
|  | 38 | H | H | Y | H | H |
|  | 39 | L | L | S | L | F/I |
|  | 168 | E | E/D | E | E | E |
|  | 170 | E | G | G | E | E |
|  | 189 | V | V | V | I | L |
|  | 191 | K | K | K | R | R |
|  | 199 | R | R | H | R | R |
|  | 200 | P | P | P | P | L |
| T cell epitopes | 66 | T | T | C | S | T |
|  | 117 | L | L | F | L | L |
|  | 121 | T | I/V | I | T | I |
|  | 124 | V | A | I | V | V |
|  | 127 | L | L | L | F | L |
|  | 128 | T | A | V | A | A |
|  | 152 | L | L | I | L | L |
|  | 161 | I | I | I | I | V |
